# Ab initio adiabatic study of the AgH system

**DOI:** 10.1038/s41598-021-87433-2

**Published:** 2021-04-15

**Authors:** Tahani A. Alrebdi, Hanen Souissi, Fatemah H. Alkallas, Fatma Aouaini

**Affiliations:** 1 Physics Department, College of Sciences, Princess Nourah Bint Abdulrahman University, P.O Box 84428, Riyadh, 11671 Saudi Arabia; 2 Laboratoire de Physique Quantique, Faculté des Sciences de Monastir, Université de Monastir, Avenue de l’Environnement 5019, Monastir, Tunisie

**Keywords:** Biophysics, Cancer, Molecular biology, Chemistry, Physics

## Abstract

In the framework of the Born–Oppenheimer (BO) method, we illustrate our ab-initio spectroscopic study of the of silver hydride molecule. The calculation of 48 electrons for this system is very difficult, so we have been employed a pseudo-potential (P.P) to reduce the big number of electrons to two electrons of valence, which is proposed by Barthelat and Durant. This allowed us to make a configuration interaction (CI). The potential energy curves (PECs) and the spectroscopic constants of AgH have been investigated for Σ^+^, Π and Δ symmetries. We have been determined the permanent and transition dipole moments (PDM and TDM), the vibrational energies levels and their spacing. We compared our results with the available experimental and theoretical results in the literature. We found a good accordance with the experimental and theoretical data that builds a validation of the choice of our approach.

## Introduction

Transition metal hydrides (TMH) play a crucial role in the chemistry due to their potential use from catalysis to energy applications^1–3^. In this context, many efforts have been carried out understand their spectroscopic, electronic, and structural properties of TMH. Among them, AgH have been studied by Stephen et al.^4^ using large valence basis sets in connective with relativistic effective core potentials (RECPs).

The silver hydride AgH molecule has been the subject of several experimental^5–11^ and theoretical^4, 12–25^ works. There is four experimental studies have been examined by Le Roy et al.^5^, Seto et al.^6^, Rolf-Dieter et al.^7^ and Helmut et al.^8^. In addition, this system has been theoretically studied by several works^4, 12–25^. Their works were only limited to the study of the ground state X^1^Σ^+^ and the first excited state A^1^Σ^+^. We have been performed a study on AgH molecule because of the absence of the characteristic spectroscopic results for the AgH molecule required for the drafting and the realization of many experimental work. Bengtsson and Olsson^26^ have been determined the first spectroscopic constants for the A^1^Σ^+^ and X^1^Σ^+^ states by determining the emission spectrum of the A^1^Σ^+^–X^1^Σ^+^ transition.

We have been beginning by determining the curves of adiabatic potential energy of all states (Σ), (Π) and (Δ) symmetries singlets and triplets that are tends to their ionic limit (Ag^+^H^−^) as well as the constants spectroscopic (well depth D_e_, equilibrium distance R_e_, transition energy vertical T_e_, the anharmonicity constant ω_e_χ_e_, the vibration pulsation ω_e_ and the constant rotational B_e_).

## Theoretical background

The spectroscopic is a main topic in the theoretical research, which is carried out in our Laboratory of Quantum Physics. We have been performed an ab-initio study of the AgH molecule in the framework of the adiabatic B.O. approximation to determine the ground state X^1^Σ^+^ and the other lowest excited states of sigma(Σ^+^), pi (Π)and delta(Δ)symmetries.

The silver atom is composed of 47 electrons whose (1S2, 2S2, 2p6, 3S2, 3p6, 3d10, 4S2, 4p6, 4d10, 5s1) is the fundamental electronic configuration. This atom is considered as a system with a one valence electron by replacing the core electrons with a proposed pseudo-potential of Barthelat and Durand^27, 28^. Whereas, the hydrogen atom is composed of one electron, when the fundamental electronic configuration is (1s^1^). The interaction of the silver core with the electrons valence of hydrogen atom is represented by the core polarization potential (CPP), giving by Muller et al.^29^ and it is given as follows

1 $${V_{CPP}} = - \frac{1}{2}\sum\limits_\gamma {{\alpha_\gamma }\overrightarrow {f_\gamma{^\prime}} \overrightarrow {f_\gamma } }$$

${\kern 1pt} {\vec f_\gamma }$ is the electrostatic field that is at center γ generated through the valence electrons and all the other centers’ cores and ${\alpha_\gamma }$ is the dipole polarizability of the core γ that is given as following.

2 $$\overrightarrow {f_\gamma } = \sum\limits_i {\frac{{{{\overrightarrow R }_{\gamma i}}}}{{R_{{\gamma}i}^3}}{F_l}({R_{\gamma i}},\rho_\gamma^l) - \sum\limits_{{\gamma{^\prime}} \ne \gamma } {{Z_c}\frac{{{{\overrightarrow R }_{\gamma {^\prime}\gamma }}}}{{R_{{\gamma{^\prime}}\gamma }^3}}} }$$

${F_1}({R_{{\gamma}i}},{\rho_\gamma })$ represents the cut-off function dependent on ρ_γ_ according to the expression given by Foucault et al.^30^ in the following form.

3 $$F({R_{\gamma i}},{\rho_\gamma }) = \left\{ \begin{gathered} 0;\,{R_{\gamma i}} < {\rho_\gamma } \hfill \\ 1;\;{R_{\gamma i}} > {\rho_\gamma } \hfill \\ \end{gathered} \right.$$

where the formulation present the cut-off radius.

$$F({R_{{\gamma}i}},{\rho_\gamma }) = \sum\limits_{l = 0}^\infty {\sum\limits_{m = - l}^{ + 1} {{F_l}({R_{{\gamma}i}},{\rho_\gamma })\left| {lm\gamma } \right\rangle \left\langle {lm\gamma } \right|} }$$

whereas, the operator $\left| {lm\gamma } \right\rangle \left\langle {lm\gamma } \right|$ was the spherical harmonic in the center of core γ.

The parameters α_γ_ and ρ_γ_ were adjusted to reproduce the experimental ionization potential and the energies of the lowest excited levels. We have been used the core polarizability of the silver is α_Ag_ = 9.32$a_0^3$^29^ and *ρ*_s_ = *ρ*_p_ = *ρ*_d_ are the optimized cut-off parameters are equal to 2.00 Bohr.

## Results

### Basis set

To have a perfect representation of this atomic levels (7s, 7p, 6d, 8s, 8p, 7d, 9s and 9p) of Ag atom, we have been optimized a large Gaussian-Type Orbital (GTO) basis set, which is 8s/6p/5d (see Table 1). While for the hydrogen atom, we have been used this basis (7s/3p/2d), which was re-optimized by the basis set studied by Zrafi et al.^31^ (see Table 2). Therefore, we have been ameliorated the difference between our data and the experimental ones^32^ that the differences are acceptable (< 33.68 cm^−1^ for silver and < 50 cm^−1^ for hydrogen) (see the Tables 1 and 2).

**Table 1:** Theoretical ionization energies (in cm^−1^) of silver atom compared with the experimental ones^32^.

Atomic levels	This work	Experimental^32^	ΔE
5 s	− 61,106.450	− 61,106.450	0.00
5p	− 30,940.655	− 30,940.655	0.00
6 s	− 18,553.068	− 18,550.298	2.80
6p	− 12,639.763	− 12,673.446	33.68
5d	− 12,350.276	− 12,350.331	0.00
7 s	− 9213.106	− 9219.476	6.40
7p	− 6985.658	− 7012.034	26.40
6d	− 6892.600	− 6897.060	4.45

*ΔE* Energy difference between the experimental values and theoretical work in cm^−1^.

**Table 2:** Theoretical ionization energies (in cm^−1^) of hydrogen atom compared with the experimental ones^32^.

Atomic levels	This work	Experimentale^32^	ΔE
1s	− 109,725.89982	− 109,678.77159	47.12
2s	− 27,371.77786	− 27,419.81712	48.04
2p	− 27,369.80259	− 27,419.60862	49.8
3p	− 12,189.62067	− 12,186.48811	3.13

*ΔE* Energy difference between the experimental values and theoretical work in cm^−1^.

We have been used a chain of programs developed in the quantum physics laboratory in Toulouse to investigate the PECs and the dipole moments. This chain is composed of the Toulouse package code (RCUT, PSHF, IJKL, FOCK, CIPSI, CVAL, MOYEN, BDAV…)^33–52^. The spectroscopic parameters were determined by fitting the vibrational levels with the method of least square. The ionization potential of silver is 61,106.45 cm^−1^ and the electron affinity of hydrogen is 6083.057 cm^−1^. The energy of the first ionic limit Ag^+^ + H^−^ is equal to 55,023.393 cm^−1^ (see Table 3).

**Table 3:** Various molecular states of AgH below the ionic limit (Ag^+^ + H^−^).

Asymptotes	Molecular states	This work (cm^−1^)	Exp^32^-PI(cm^−1^)	ΔE
Ag (5 s) + H (1 s)	${}^{1,3}\Sigma$	− 170,785.2216	− 170,785.2216	0.00
Ag (5p) + H (1 s)	${}^{1,3}\Sigma$,${}^{1,3}\Pi$	− 140,619.4266	− 140,619.4266	0.00
Ag (6 s) + H (1 s)	${}^{1,3}\Sigma$	− 128,278.9678	− 128,229.0696	49.9
Ag (6p) + H (1 s)	${}^{1,3}\Sigma$,${}^{1,3}\Pi$	− 122,365.6628	− 122,352.2176	13.45
Ag (5d) + H (1 s)	${}^{1,3}\Sigma$,${}^{1,3}\Pi$,${}^{1,3}\Delta$	− 122,029.1026	− 122,029.1026	0.00
Ag (7 s) + H (1 s)	${}^{1,3}\Sigma$	− 118,939.0058	− 118,898.2476	40.76
Ag (7p) + H (1 s)	${}^{1,3}\Sigma$,${}^{1,3}\Pi$	− 116,711.5578	− 116,690.8056	20.76
Ag (6d) + H (1 s)	${}^{1,3}\Sigma$,${}^{1,3}\Pi$,${}^{1,3}\Delta$	− 116,618.4998	− 116,575.8316	42.67

*ΔE* Energy difference between the experimental values and theoretical work in cm^−1^.

### Adiabatic PEC_S_ and their spectroscopic parameters

**Figure 1:**
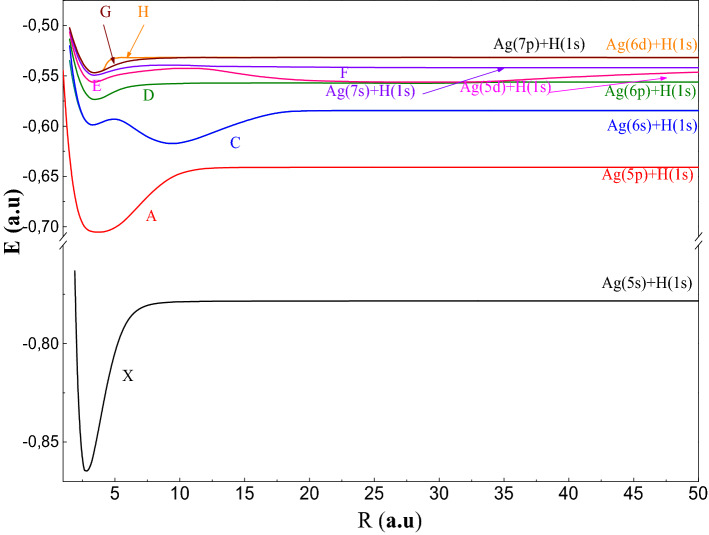
Potential energy curves of the ^1^Σ^+^ states of AgH molecule.

**Figure 2:**
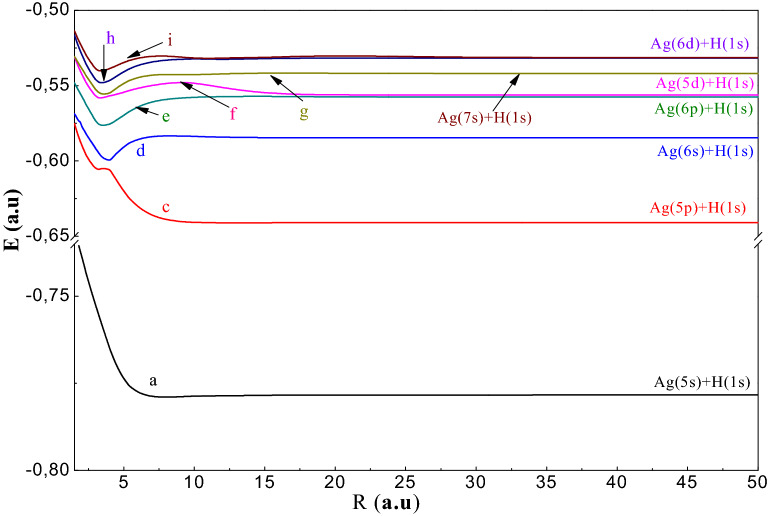
Potential energy curves of the ^3^Σ^+^ states of AgH molecule.

**Figure 3:**
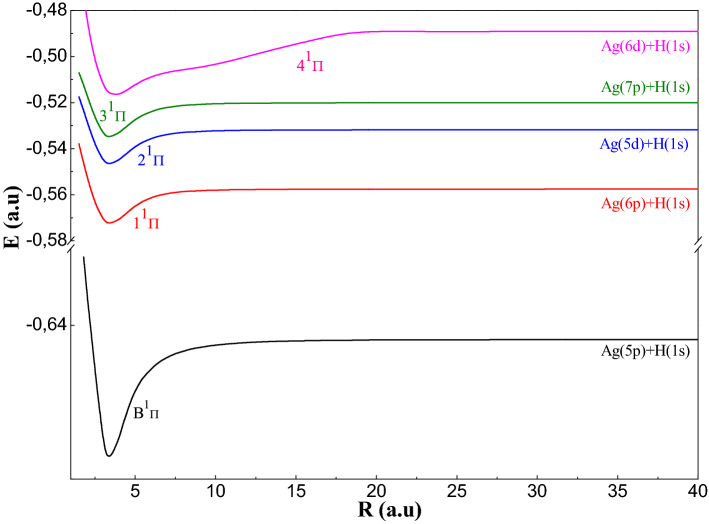
Potential energy curves of the ${}^1\Pi$ states of AgH molecule.

**Figure 4:**
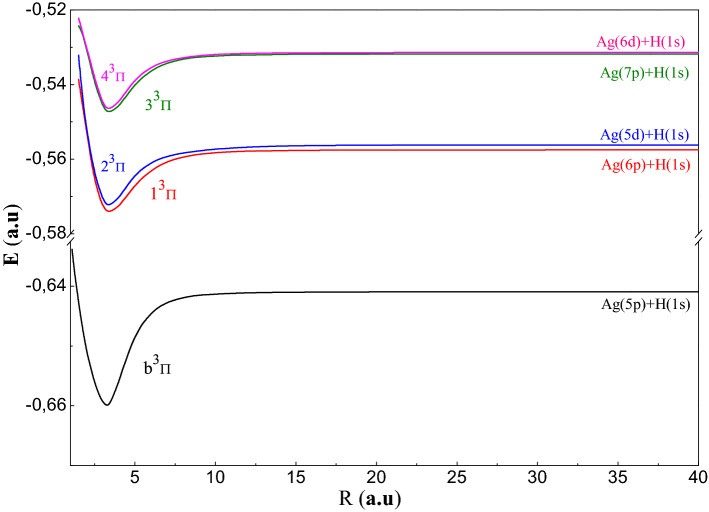
Potential energy curves of the ${}^3\Pi$ states of AgH molecule.

**Figure 5:**
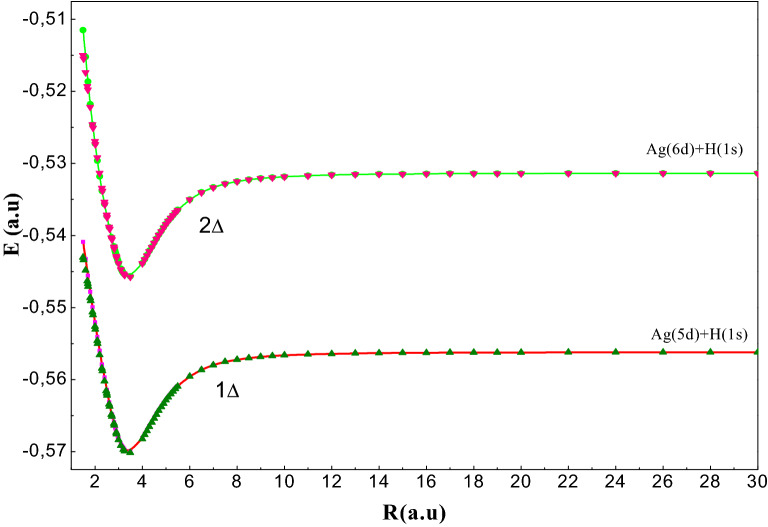
Potential energy curves of the ^1^Δ (symbol) and ^3^Δ (continuous line)states of AgH molecule.

To study the AgH molecule we have been used the P.P approach, which reduces the number of electrons in the molecular system to two valence electrons which allows, thereafter, performing a complete configuration interaction. In this part, we have been displayed the adiabatic results: PECs and spectroscopic constants (R_e_: equilibrium distance, De: well depth, T_e_: excitation energy vertical, ω_e_: the pulsation at equilibrium, ω_e_χ_e_: the constant of anharmonicity and B_e_: the constant rotational) of the 30 electronic states ^1,3^Σ^+^, ^1,3^Π and ^1,3^Δsymmetries tends to the ionic limit (Ag^+^ + H^−^). In Figs. 1, 2, 3, 4, 5, we have been drawn these curves for a huge grid of points from 1.5to 200 a.u. In Table 4, we have been displayed the spectroscopic parameters states’ with the available theoretical work.

**Table 4:** Spectroscopic constants for ^1,3^Σ^+^, ^1,3^Π and ^1,3^Δ states of AgH.

States	R_e_ (a.u.)		D_e_ (cm^-1^)		T_e_ (cm^−1^)	ω_e_ (cm^−1^)	ω_e_χ_e_ (cm^−1^)		B_e_ (cm^−1^)	References
**(a) **^**1**^**Σ**^**+**^** states**										
X^1^Σ^+^	2.91		19,100		0	1606	27		7.7	This work
	3.05		19,250 (± 200)		0	–	–		–	le Roy^5^
	3.04		19,300		–	–	–		–	Seto^6^
	3.05		21,200		0	1759.9	34.06		6.45	Witek^12^
	3.04		–		0	1819	80		6.36	Li^13^
	2.95		20,387		–	2073	52.7		6.904	Witek^14^
	3.06		–		–	1760	–		6.43	Brike et al.^8^
A^1^Σ^+^	3.35		17,989		35,634	1376	28		4.38	This work
	3.11		18,830.646		29,971	–	–		–	le Roy^5^
	3.15		19,100		29,959	1663.6	87		6.265	Witek^12^
	3.09		–		30,321	–	–		6.17	Li^13^
	3.24		18,544		32,208	1422	27.3		5.730	Witek^14^
	3.03		–		–	1805	–		6.56	Brike et al.^8^
C^1^Σ^+^	3.32		3154		58,968	–	2.63		5.47	This work
2nd min	9.45		7233		–	303	–		0.68	
D^1^Σ^+^	3.32		3154		58,999	1665	75		5.47	
2nd min	9.44		7233		–	–	–		–	
E^1^Σ^+^	3.5		3852		64,958	719	36		4.92	
F^1^Σ^+^	3.4		3178		68,443	23	0.74		5.2	
**(b) **^**3**^**Σ**^**+**^** states**										
a^3^Σ^+^		7.87	121	57,404		75	12		0.97	This work
c^3^Σ^+^		3.21	22	60,476		17	3.62		5.85	
d^3^Σ^+^		3.85	3494	64,517		929	61.26		4.07	
e^3^Σ^+^		3.58	4208	67,987		628	23.09		4.7	
f^3^Σ^+^		3.36	457	68,787		564	22.06		5.34	
2^nd^min		27.6	3	–		–	–		0.079	
g^3^Σ^+^		3.64	3108	69,007		708	40.63		4.55	
h^3^Σ^+^		3.48	3629	70,458		617	26.78		4.98	
i^3^Σ^+^		3.41	1990	71,983		653	52.05		5.18	
**(c) **^**1,3**^**Π states**										
B^1^Π		3.42	1700		35,756	422	27.81		5.15	This work
b^3^Π		3.21	4244		45,210	651	24.92		5.85	
1^1^Π		3.46	3282		65,094	660	33.75		5.04	
1^3^Π		3.45	3647		64,594	583	23.73		5.06	
2^1^Π		3.45	3269		70,706	610	28.8		5.06	
2^3^Π		3.42	3546		65,061	558	23.07		5.15	
3^1^Π		3.44	3278		73,269	624	30.17		5.09	
3^3^Π		3.45	3437		70,502	599	26.56		5.06	
4^1^Π		3.73	6093		78,205	346	7.87		4.33	
4^3^Π		3.43	3336		70,693	600	27.7		5.12	
**(d) **^**1,3**^**Δ States**										
1^1^Δ		3.43	3197		65,500	700	38.49	5.12		This work
1^3^Δ		3.42	3069		65,456	631	32.28	5.15		
2^1^Δ		3.43	3030		70,862	570	26.26	5.12		
2^3^Δ		3.43	3169		70,840	634	31.65	5.12		
3^1^Δ		3.43	3155		73,329	609	29.6	5.12		
3^3^Δ		3.44	3205		73,318	634	31.42	5.09		

In Fig. 1, we present the adiabatic PECs of the states of^1^Σ^+^ symmetry of the AgH molecule over the inter-nuclear distance interval R between 1.5 a.u and 50 a.u. We can see in this figure that the ground state X^1^Σ^+^ dissociates towards their asymptotic limit (Ag (5s) + H (1s)) and has a single deep well (D_e_ = 19,100 cm^−1^), which is near of reference^1^ (D_e_ = 19,250 ± 200 cm^−1^).Our equilibrium distance is of the order of 2.91 a.u, which is near to the equilibrium distance R_e_ = 2.95 a.u^5^. Moreover, our pulsation ω_e_ is equal to 1606 cm^−1^ and our anharmonicity constant ω_e_χ_e_ = 27 cm^−1^ are near to that obtained by Witek et al.^14^ (= 1759.9 cm^−1^ and ω_e_χ_e_ = 34.06 cm^−1^). Turn on the first excited A^1^Σ^+^ state, which dissociates towards Ag (5p) + H (1s) has a wider well (De = 17,989 cm^−1^) at R_e_ = 3.35a.u.Then, the second excited state C^1^Σ^+^ tends rapidly towards their dissociation limit (Ag (6s) + H (1s)) at the distance 25 a.u. Indeed, C^1^Σ^+^ have double well, the first well is of depth 3154 cm^−1^ at R_e_ = 3.32 a.u. and the second is of depth of 7233 cm^−1^ at R_e_ = 9.45 a.u. We present in Table 4a the comparison of our spectroscopic parameters with that available in the literature^5, 6, 8, 12–14^ for the states of X^1^Σ^+^ and A^1^Σ^+^. We notice that the difference between the well depth of X^1^Σ^+^ for Le Roy el al.^5^, Seto et al.^6^ and Witek et al.^12^ is of the order of 2000 cm^−1^. On the other hand, the difference between our well depth and those for Le Roy el al.^5^ and Seto et al.^6^ is of the order of 150 cm^−1^. Concerning the equilibrium distance, the difference between our result and that for Witek et al.^14^ is equal to 0.04 a.u. In addition, the comparison between our results of A^1^Σ^+^ and that in the literature is in good accordance (see Table 4a). The PECs of the higher excited states of symmetry sigma singlets denoted D; E; F; G and H are presented in the same Fig. 1. All of these states are attractive and their well depths are not deep. We observe the existence of the avoided crossings between the states of the same nature (neutral–neutral) at short distance [(D^1^Σ^+^, E^1^Σ^+^) and (E^1^Σ^+^, F^1^Σ^+^)] and ionic neutral [(X^1^Σ^+^, A^1^Σ^+^), (A^1^Σ^+^, C^1^Σ^+^) and (C^1^Σ^+^, D^1^Σ^+^)] (see Table 5). These crossings become less and less avoided, and the difference of energy at these positions becomes smaller at long distances. Their spectroscopic parameters are given in Table 4, which are determined for the first time.

**Table 5:** Avoided crossing positions.

States	X /A	A/C	C/D	D/E	E/F	F/G	G/H
Positions (a.u)	5	9	17	34	35	64	181.6

We have been presented in Fig. 2, the adiabatic potential energy curves of the triplet sigma states ^3^Σ^+^. We notice that a^3^Σ^+^ and c^3^Σ^+^ are almost repulsive because of the lack of interaction with the ionic curve except for the state d, e and h which present a shallow well of values 3494, 4208 and 3629 cm^−1^, respectively. The spectroscopic parameters of these states are given in Table4b.

We have been displayed in Figs. 3 and 4, the PECs related respectively to the ^1,3^Π symmetry states. These curves relating to these states have a regular shape. Indeed, all the curves have a single minimum of potential and tend quickly (≈ 15 a.u.) towards their asymptotic limit except of state 4^1^Π. The triplet states^3^Π are deeper than the singlet ones (De > 1700 cm^−1^ for ^1^Π and De > 3336 cm^−1^ for ^3^Π). The spectroscopic parameters of the ^1,3^Πstates are given in Table 4c. We notice that in Fig. 4 that the states (1^3^Π, 2^3^Π) and (3^3^Π, 4^3^Π) have avoided crossings between them.

We have been studied four states of delta 4^1,3^Δ symmetry. The PECs related to the 4^1,3^Δ states are drawn respectively in Fig. 5. These curves have regular shapes with one minimum potential of the order of 3.43 a.u. Moreover, the triplets and singlets^1,3^Δ dissociating towards the same limit are quasi-degenerate which is confirmed by the results in Table 4d.

### Electronic dipole moment properties of AgH

**Figure 6:**
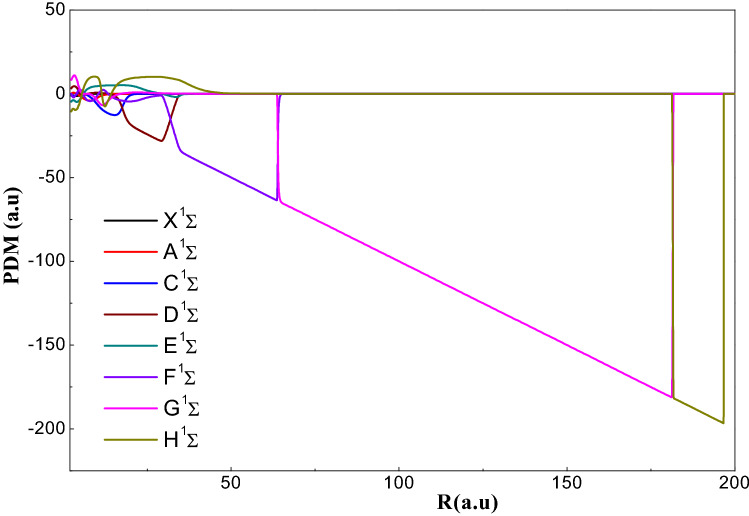
Permanent dipole moment for the ^1^Σ^+^ states for the AgH molecule.

In Fig. 6, we have been displayed the curves of the permanent dipole moments PDM of (1–8) ^1^Σ^+^of AgH. We can see that all the curves has a linear part and all of these linear form are segments of the identical line of slope (−R). We can see that the junction between two linear forms belonging to two successive states ^1^Σ^+^ corresponding to an avoided crossing between the states. Therefore, that at the avoided crossing there is a sudden variation in the permanent dipole moment, and this inter-nuclear distance becomes bigger. On the other hand, that the dipole moment variation at short range is very smooth. There are slow variations at short inter-nuclear distance although this variation is sharp for large distances (see Figure S1.a and b).

**Figure 7:**
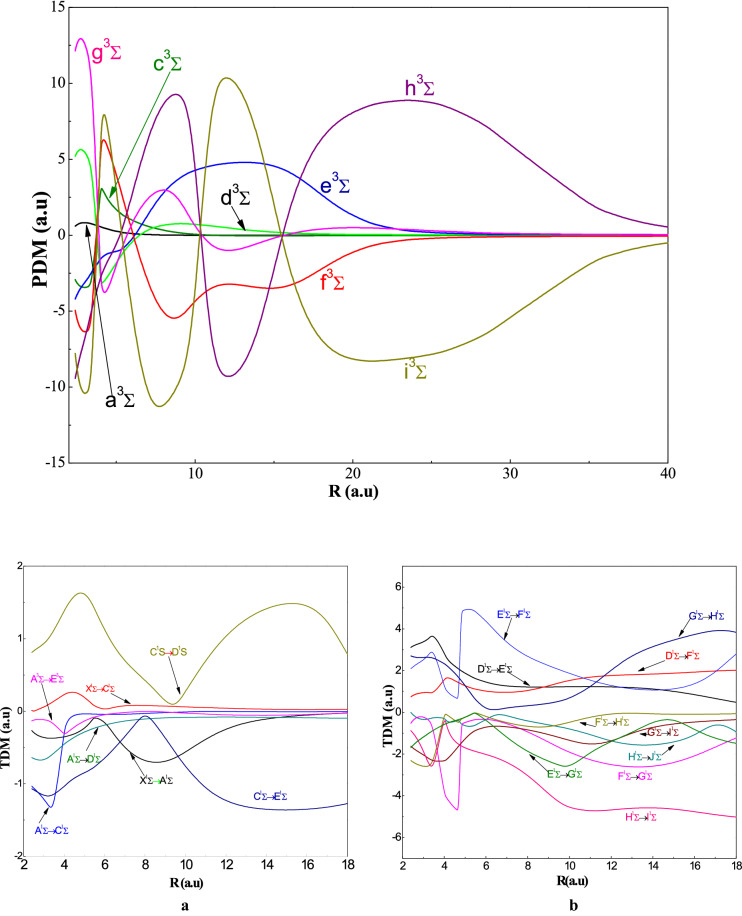
Permanent dipole moment for the ^3^Σ^+^ states for the AgH molecule. (**a**) Transition Dipole Moment from the states i = 1, 2 et 3 to the states j = i + 1,i + 2 of the symmetry ${}^1\Sigma$ of AgH molecule. (**b**) Transition Dipole Moment from the states i = 4, 5, 6,7 et 8 to the states j = i + 1,i + 2 of the symmetry ${}^1\Sigma$ of AgH molecule.

The curves of the PDM of the 8 triplet sigma states ^3^Σ^+^are shown in Fig. 7 whose the inter-nuclear distance varies from 2 to 60 (a.u.). From this figure, we can observe that there is no abrupt variation and there is no line segments associated with the ionic character as in symmetry ^3^Σ^+^. The analysis of these curves shows the presence of short-distance extreme, which is explained by the transfer of charges between neutral states. This is justifies the absence of the ionic curve. We can observe in Figure S2 that the crossings of the PDM variation curves from ^1^Π states for R = 9 a.u. and R = 18 a.u. related to the positions of the avoided crossings (P.A.C). Whereas, for the short distance, the variation is slight and for the long distance this variation is abrupt. We can see that the curves of the singlet states are identical to those of the triplet states that confirm the shapes of the PECs (see Figure S3).

**Figure 8:**
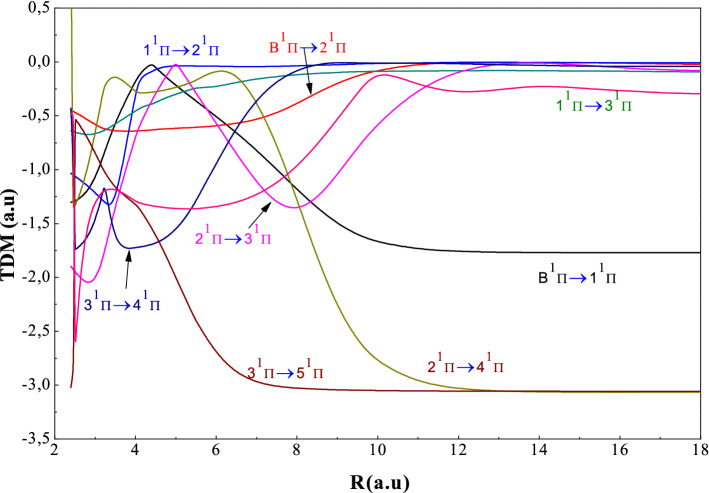
Transitiondipole moment for the ^1^Πstates for the AgH molecule.

**Figure 9:**
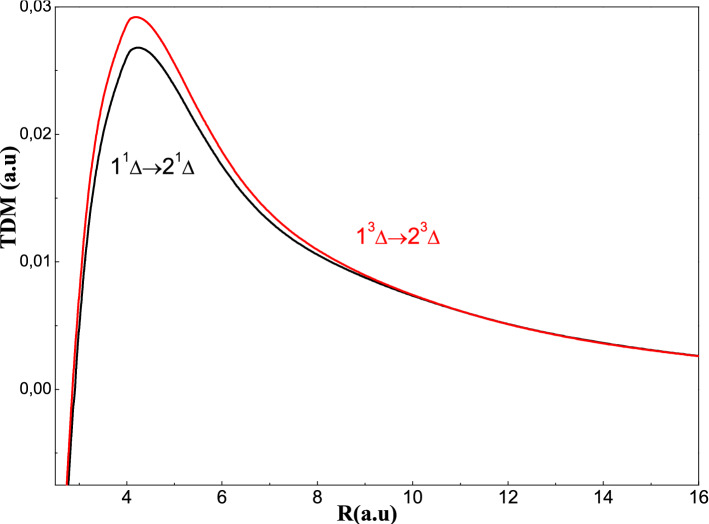
Transitiondipole moment for the ^1^Δstates for the AgH molecule.

Move on for the transition dipole moment curves, we observe in Fig. 7a,b that the variations are slight and the extremes coincide at the P_AC_ in the PECs for example, the X-A transitions at a maximum of the order of 5 a.u. and the C-D transitions at a maximum of the order of 4.5 a.u. So, we can conclude that these extremes are characterized by the maximum of ionic character. The observation of Fig. 8 shows that the variations are slight as the curves of the TDMs correspond to ^1^Σ^+^ symmetry and the maximum of TDM (2^1^Π-3^1^Π) is located at R = 5.2 a.u. corresponding to the P.A.C. in the potential energy curves. Examining the Fig. 9, we find that these transitions of ^1,3^Δ have slight variations; passing through a single extreme corresponding to the maximum of ionic character.

### Vibrational levels of AgH

After determining of the dipole moments, we have been investigated the vibrational levels of 30 electronic states as well as their spacing’s. The analysis of the vibrational levels of different electronic states is of great importance. Indeed, the spacing between these vibration levels provides precise information on the shapes of the PECs as indicated in reference^35^.

**Figure 10:**
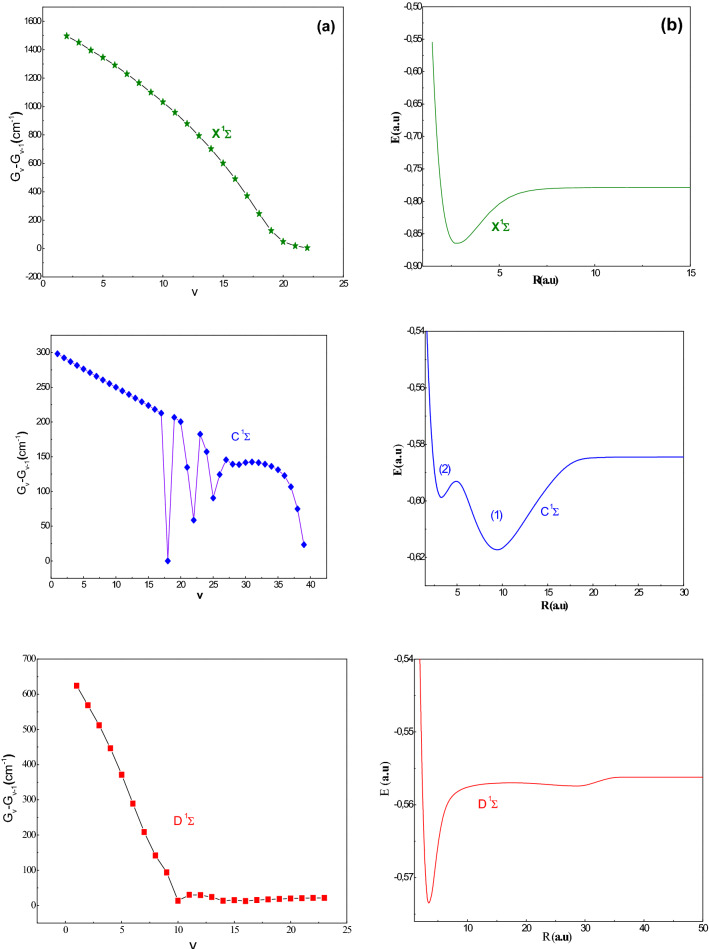
Vibrational spacing (left) and potential energy curves (right) for (X, C and D) ^1^Σ^+^ states of AgH.

In Fig. 10, we have been illustrated the spacing’s between the vibrational levels of the ground state X^1^Σ^+^. Note that the spacing’s are not constant, which is reflected the anharmonicity of the well. At the beginning, the variation is linear decreasing which related to the regular anharmonic shape of the PECs (see Fig. 10a) then it is constant and attaints the dissociation limit Ag (5p) + H (1 s) (see Fig. 10b). This behavior is similar to the ground state spacing of BaH^+^^35^ and X^2^Σ^+^ barium hydride from BaXe^36^. The observation of the state C^1^Σ^+^ indicate the variation of the curve of spacing’s between levels is almost linear and decreasing until v = 17 and we have a degenerate level v = 18 therefore the appearance of a second well smaller than the other. Then, from v = 27 to v = 40, we observe a significant drop which reflects the presence of very numerous and tight spaces near to the limit. Move on to the D^1^Σ^+^, we can see the shape of the well is regular anharmonic up to v = 10 as a result of a sudden change of pace which translates an important and rapid widening, from v = 10, the levels become tight and numerous.

## Conclusion

We have been started this work by building and optimizing the bases to reproduce the transition energy spectra of silver atoms and hydrogen. Next, we have been calculated the adiabatic PECs of 30 molecular states (16 ^1,3^Σ^+^, 10 ^1,3^Π, and 4 ^1,3^Δ) lying to the Ag^+^ + H^−^ asymptotic limit. Then, we have been calculated the spectroscopic parameters (D_e_, R_e_, T_e_, ω_e_χ_e_, ω_e_ and B_e_) from these curves. We compared our study with experimental and theoretical ones available in the literature^6–25^. We observe a good accordance with the experimental and theoretical data, which builds a validation criterion for our method.

We have been determined the vibration levels of each electronic state as well as their spacing’s. Analysis of these properties gives precise information about the shape of the PECs. Moreover, we have been investigated the electrical dipolar properties (PDM, TDM), which make it possible to confirm that the ionic character for the AgH molecule is in the ^1^Σ^+^ symmetry.

## Supplementary Information

Supplementary Information

## References

[1] Evgeny L, Houhua L, Clément M (2014). Well-defined transition metal hydrides in catalytic isomerizations. Chem. Commun..

[2] Tianfei L, Meiyuan G, Or A, Reiner L, Marcus L, Sascha O, Leif H (2018). Accelerating proton-coupled electron transfer of metal hydrides in catalyst model reactions. Nat. Chem..

[3] Abdullah C, Mustafa K (2018). Electronic structure, elastic and phonon properties of perovskite-type hydrides MgXH3 (X = Fe, Co) for hydrogen storage. Solid State Commun..

[4] Langhoff SR, Pettersson LGM, Bauschlicher CW, Partridge H (1987). Theoretical spectroscopic parameters for the low-lying states of the second-row transition metal hydrides. J. Chem. Phys..

[5] Le Roy RJ, Appadoo DRT, Anderson K, Shayesteh A (2005). Direct-potential-fit analysis of new infrared and UV/visible A^1^Σ^+^-X ^1^Σ^+^ emission spectra of AgH and AgD. J. Chem. Phys..

[6] Seto JY, Morbi Z, Charron F, Lee SK, Bernath PF, Le Roy RJ (1999). Vibration-rotation emission spectra and combined isotopomer analyses for the coinage metal hydrides: CuH & CuD, AgH & AgD, and AuH & AuD. J. Chem. Phys..

[7] Rolf-Dieter U, Birk H, Polomsky P, Jones H (1991). The ground state infrared spectra of four diatomic deuterides (GaD, InD, TlD, AgD) and the determination of mass-independent molecular parameters. J. Chern. Phys..

[8] Brike H, Jones H (1989). The ground state infrared spectra of two isotopic forms of silver hydride (107AgH and 109AgH). Chem. Phys. Lett..

[9] Holden SJ, Rossington DR (1964). Hydrogen adsorption on silver, gold, and aluminum. Studies of parahydrogen conversion. J. Phys. Chem..

[10] Souissi H, Mohamed BY (2021). Application of innovative analytical modeling for the physicochemical analysis of adsorption isotherms of silver nitrate on helicenes: Phenomenological study of the complexation process. Adsorp. Sci. Technol. Article ID.

[11] Manel BY, Mohamed BY (2020). Physico-chemical study of complexation of silver ion (Ag+) by macrocyclic molecules (hexa-Helicenes) based on statistical physics theory: New description of a cancer drug. Sci. Rep..

[12] Witek HA, Nakajima T, Hirao K (2000). Relativistic and correlated all-electron calculations on the ground and excited states of AgH and AuH. J. Chem. Phys..

[13] Li Y, Libermann HP, Buenker RJ, Pichl L (2004). A coupled treatment of ^1^Σ^+^ and ^3^π states of AgH molecule. Chem. Phys. Lett..

[14] Witek HA, Fedorov DG, Hirao K, Viel A, Widmark PO (2002). Theoretical study of the unusual potential energy curve of the A 1Σ+A 1Σ+ state of AgH. J. Chem. Phys..

[15] Richard Martin L., All-Electron Relativistic Calculations on AgH. An Investigation of the Cowan-Griffin Operator in a Molecular Species, J. Phys. Chem., **87**, 750–754 (1983).

[16] Robert Le Roy J, Huang Y (2002). Representing Born-Oppenheimer breakdown radial correction functions for diatomic molecules. J. Mol. Struct. (Theochem).

[17] Mohanty AK, Parpia FA (1996). Fully relativistic calculations for the ground state of the AgH molecule. Phys. Rev. A.

[18] Jenning SY, Zulfikar M, Frank C, Lee SK, Bernath PF, Le Roy RJ (1999). Vibration-rotation emission spectra and combined isotopomer analyses for the coinage metal hydrides: CuH & CuD, AgH & AgD, and AuH & AuD. J. Chem. Phys..

[19] Liu CW, Chang H-W, Sarkar B, Jean-Yves S, Samia K, Ying-Yann W (2010). Stable silver(I) hydride complexes supported by diseleno-phosphate ligands. Inorg. Chem..

[20] Hess BA, Chandra P (1987). Relativistic *ab initio* CI study of the *X*^1^Σ^+^ and *A*^1^Σ^+^ states of the AgH molecule. Phys. Script..

[21] Andrews L, Wang X (2003). Infrared spectra and structures of the stable CuH_2_^−^, AgH_2_^−^, AuH_2_^−^, and AuH_4_^−^ anions and the AuH_2_ molecule. J. Am. Chem. Soc..

[22] Xie H, Xi X, Liu Z, Cong R, Qin Z, Xi W, Zi T, Fan H (2012). Probing the structural and electronic properties of Ag_*n*_H^−^ (*n* = 1–3) using photoelectron imaging and theoretical calculations. J. Chem. Phys..

[23] Charlene CL, Kenneth DG, Henry SF (1995). Relativistic and correlation effects in CuH, AgH, and AuH: Comparison of various relativistic methods. J. Chem. Phys..

[24] Richard RB, Walter EC (1985). Ab initio calculations including relativistic effects for Ag^2^, Au^2^, AgAu, AgH, and AuH. J. Phys. Chem..

[25] Stoll H, Fuentealba P, Dolg M, Flad J, Szentpály L, Preuss H (1983). Cu and Ag as one-valence-electron atoms: CI results and quadrupole corrections for Cu2, Ag2, CuH, and AgH. J. Chem. Phys..

[26] Bengtsson E, Olsson E (1931). Eine neue Untersuchung über die Banden des Silberhydrides. Z. Phys..

[27] Durand Ph, Barthelat JC (1975). A theoretical method to determine atomic pseudopotentials for electronic structure calculations of molecules and solids. Theor. Chim. Acta.

[28] Durand Ph, Barthelat JC (1974). New atomic pseudopotentials for electronic structure calculations of molecules and solids. Chem. Phys. Lett..

[29] Müller W, Flesh J, Meyer W (1984). Treatment of intershell correlation effects in ab initio calculations by use of core polarization potentials. Method and application to alkali and alkaline earth atoms. J. Chem. Phys..

[30] Foucrault M, Millie P (1992). Nonperturbative method for core–valence correlation in pseudopotential calculations: Application to the Rb_2_ and Cs_2_ molecules. J. Chem. Phys..

[31] Zrafi W, Oujia B, Gadea FX (2006). Theoretical study of the CsH molecule: adiabatic and diabatic potential energy curves and dipole moments. J. Phys. B: At. Mol. Opt. Phys..

[32] Ralchenko, Y., Kramida, A., Reader, J., NIST ASD Team 2011 NIST Atomic Spectra Database (version 4.1). Available at: http://physics.nist.gov/asd/.

[33] Gaied W, Habli H, Oujia B, Gadea FX (2011). Theoretical study of the MgAr molecule and its ion Mg^+^Ar: Potential energy curves and spectroscopic constants. J. Eur. Phys..

[34] Gaied W, Oujia B (2010). Potential energy curves, permanent and transition dipole moments for numerous electronic excited states of CaAr. Int. J. Nanoparticles.

[35] Mejrissi L, Habli H, Ghalla H, Oujia B, Gadea FX (2013). Adiabatic ab initio study of the BaH^+^ ion including high energy excited states. J. Phys. Chem. A.

[36] Abdesslem K, Mejrissi L, Issaoui N, Oujia B, Gadéa FX (2013). One and two-electron investigation of electronic structure for Ba^+^Xe and BaXe van der Waals molecules in a pseudopotential approach. J. Chem. Phys..

[37] Habli H, Ghalla H, Oujia B, Gadea FX (2011). Ab initio study of spectroscopic properties of the calcium hydride molecular ion. Eur. Phys. J. D.

[38] Habli H, Dardouri R, Oujia B, Gadéa FX (2011). Ab initio adiabatic and diabatic energies and dipole moments of the CaH^+^ molecular ion. J. Chem. Phys..

[39] Chaieb M, Habli H, Mejrissi L, Oujia B, Gadea FX (2014). Ab initio spectroscopic study for the NaRb molecule in ground and excited states. J. Quantum Chem..

[40] Habli H, Mejrissi L, Issaoui N, Yaghmour SJ, Oujia B, Gadea FX (2015). Ab initio calculation of the electronic structure of the strontium hydride ion (SrH^+^). Int. J. Quantum Chem..

[41] Guérout R, Aymar M, Dulieu O (2010). Ground state of the polar alkali-metal-atom–strontium molecules: Potential energy curve and permanent dipole moment. Phys. Rev. A.

[42] Dardouri R, Issa K, Oujia B, Gadéa F (2012). Xavier, Theoretical study of the electronic structure of LiX and NaX (X = Rb, Cs) molecules. Int. J. Quant. Chem..

[43] Dardouri R, Habli H, Oujia B, Gadéa FX (2012). Theoretical study of the electronic structure of KLi molecule: Adiabatic and diabatic potential energy curves and dipole moments. Chem. Phys..

[44] Khémiri N, Dardouri R, Oujia B, Gadea FX (2013). Ab initio investigation of electronic properties of the magnesium hydride molecular ion. J. Phys. Chem. A..

[45] Khelifi N, Oujia B, Gadea FX (2007). Dynamic couplings, radiative and nonradiative lifetimes of the A^1^Σ^+^ and C^1^Σ^+^ states of the KH molecule. J. Phys. Chem. Ref. Data..

[46] Dardouri R, Habli H, Oujia B, Gadéa FX (2013). Ab Initio Diabatic energies and dipole moments of the electronic states of RbLi molecule. J. Comp. Chem..

[47] Habli H, Mejrissi L, Ghalla H, Yaghmour SJ, Oujia B, Gadéa FX (2016). Ab initio investigation of the electronic and vibrational properties for the (CaLi)^+^ ionic molecule. Mol. Phys..

[48] Souissi H, Mejrissi L, Habli H, Al-Ghamdi At A, Oujia B, Gadea FX (2017). Spectroscopic ab initio investigation of the electronic properties of (SrK)^+^. Chem. Phys..

[49] Souissi H, Jellali S, Chaieb M, Habli H, Oujia B, Gadéa FX (2017). An adiabatic spectroscopic investigation of the CsRb system in ground and numerous excited states. J. Qu. Spect. Rad. Tran..

[50] Mtiri S, Mejrissi L, Habli H, Al-Ghamdi AA, Oujia B, Gadéa FX (2017). Theoretical investigation of the diatomic Van der Waals systems Ca^+^He and CaHe. Comput. Theo. Chem..

[51] Hamdi R, Abdessalem K, Dardouri R, Al-Ghamdi At A, Oujia B, Gadéa FX (2017). Spectroscopic and electric dipole properties of Sr^+^Ar and SrAr systems including high excited states. J. Phys. B At. Mol. Opt. Phys..

[52] Souissi H, Mejrissi L, Habli H, Alsahhaf M, Oujia B, Xavier GF (2020). Ab initio diabatic and adiabatic calculations for francium hydride FrH. J. N. Chem..

