# Supplementary material for: Ab initio adiabatic study of the AgH system
Source: Sci Rep. 2021 Apr 15;11:8277. doi: 10.1038/s41598-021-87433-2 (PMC8050250; doi:10.1038/s41598-021-87433-2)
Supplement: Supplementary file 1 — Supplementary Information [file 41598_2021_87433_MOESM1_ESM.docx]

| **Figure S1.a:**Permanent dipole moment for the X, A and C^1^Σ^+^ statesofAgH molecule. | **Figure S1.b :** Permanent dipole moment for the D, E and F^1^Σ^+^ states of AgH molecule |
| --- | --- |

**Ab initio adiabatic study of the AgH system**

Tahani A. Alrebdi^a^, Hanen Souissi^b,*^, Fatemah H.Alkallas^a^, Fatma Aouaini^a^

^a^ Physics Department, College of Sciences, Princess Nourah Bint Abdulrahman University, P.O Box 84428, Riyadh 11671, Saudi Arabia.

^b^ Laboratoire de Physique Quantique, Faculté des Sciences de Monastir, Université de Monastir, Avenue de l’Environnement 5019, Monastir, Tunisie,

**(*) E-mail :**hanensouissi9@gmail.com

**Figure S2:** Permanent dipole moment for ^1^Πstates of AgH molecule.

**Figure S3 :**Permanent dipole moment for ^1^Δ(line) and ^3^Δ(symbol) states of AgH molecule.
